# Small Molecule Inhibition of Ligand-Stimulated RAGE-DIAPH1 Signal Transduction

**DOI:** 10.1038/srep22450

**Published:** 2016-03-03

**Authors:** Michaele B. Manigrasso, Jinhong Pan, Vivek Rai, Jinghua Zhang, Sergey Reverdatto, Nosirudeen Quadri, Robert J. DeVita, Ravichandran Ramasamy, Alexander Shekhtman, Ann Marie Schmidt

**Affiliations:** 1 Diabetes Research Program, Department of Medicine, New York University Langone Medical Center, 550 First Avenue, New York, 10016 New York, USA; 2 Department of Chemistry, University at Albany, State University of New York, 1400 Washington Avenue, Albany, 12222 New York, USA; 3 RJD Medicinal Chemistry and Drug Discovery Consulting LLC, 332 W. Dudley Avenue, Westfield, New Jersey 07090, USA

## Abstract

The receptor for advanced glycation endproducts (RAGE) binds diverse ligands linked to chronic inflammation and disease. NMR spectroscopy and x-ray crystallization studies of the extracellular domains of RAGE indicate that RAGE ligands bind by distinct charge- and hydrophobicity-dependent mechanisms. The cytoplasmic tail (ct) of RAGE is essential for RAGE ligand-mediated signal transduction and consequent modulation of gene expression and cellular properties. RAGE signaling requires interaction of ctRAGE with the intracellular effector, mammalian diaphanous 1 or DIAPH1. We screened a library of 58,000 small molecules and identified 13 small molecule competitive inhibitors of ctRAGE interaction with DIAPH1. These compounds, which exhibit *in vitro* and *in vivo* inhibition of RAGE-dependent molecular processes, present attractive molecular scaffolds for the development of therapeutics against RAGE-mediated diseases, such as those linked to diabetic complications, Alzheimer’s disease, and chronic inflammation, and provide support for the feasibility of inhibition of protein-protein interaction (PPI).

The Receptor for Advanced Glycation Endproducts (RAGE) is a multi-ligand receptor of the immunoglobulin superfamily of cell surface molecules^1^,^2^,^3^,^4^. The extracellular part of RAGE is composed of three immunoglobulin-like domains, V, C1 and C2, followed by a single transmembrane spanning domain and a short cytoplasmic domain^5^,^6^,^7^,^8^. The cytoplasmic domain of human RAGE, ctRAGE, is highly charged and composed of 43 amino acids (LWQRRQRRG EERKAPENQE EEEERAELNQ SEEPEAGESS TGGP)^5^. Ligand stimulation of RAGE activates signal transduction pathways, such as the mitogen activated protein kinases (MAPK); Rho GTPases; and phosphatidylinositol 3-kinase (PI3K)/Akt, in a manner dependent on cell type and the acuteness versus chronicity of the inciting signal^6^,^7^,^8^,^9^,^10^,^11^,^12^,^13^,^14^,^15^,^16^. ctRAGE is essential for RAGE signal transduction; *in vitro* and *in vivo* experiments in which this domain of the receptor was deleted revealed it was critical for transmitting the downstream effects initiated by RAGE ligands^17^.

We previously probed the proximate mechanisms by which ctRAGE exerted these effects on ligand-stimulated signaling using a yeast two-hybrid analysis and identified that ctRAGE interacted with the FH1 domain (formin homology domain 1) of mammalian form of diaphanous 1 (DIAPH1)^11^,^18^,^19^. Co-immunoprecipitation and immunolocalization experiments verified this interaction in cellular models. Small interference (si) RNA-mediated reduction of DIAPH1 expression, but not scramble control siRNAs, blocked the effects of RAGE ligands such as carboxy methyl lysine advanced glycation endproducts (CML-AGEs) and S100/calgranulins^20^,^21^ on cellular signaling in diverse cell types, including vascular cells, immune cells, cardiomyocytes and transformed cells^11^,^16^,^22^,^23^. *In vivo*, in bone marrow derived macrophages and vascular smooth muscle cells (SMCs) devoid of *Diaph1* (gene encoding DIAPH1), RAGE ligands failed to initiate cellular signaling^16^,^23^. In contrast, cellular stimuli, which are not RAGE ligands, such as platelet derived growth factor (PDGF)-BB, stimulated activation of Akt cellular signaling, migration and proliferation of SMCs in the face of reduced DIAPH1 expression^16^. These data suggested that knock-down of DIAPH1 expression did not impart generalized and non-specific suppression of intracellular effector pathways.

Based on these data indicating that DIAPH1 was required for RAGE signal transduction, solution NMR spectroscopy was used to identify interaction surfaces between ctRAGE and DIAPH1 FH1 domain. Mapping the observed chemical shift changes onto the molecular surface of ctRAGE revealed that the interaction surface between RAGE cytoplasmic domain and FH1 of DIAPH1 consists of a small positively charged patch formed by Q3, R4, R5, and Q6 with the total area less than 200 Å^2^ 24. When R6/Q6 were mutated to alanine residues, primary murine SMCs incubated with RAGE ligand S100B or CML-AGE displayed significantly reduced signaling (phosphorylation of Akt) and SMC migration and proliferation vs. vector control or wild-type RAGE. PDGF-BB, not a RAGE ligand, initiated signaling and triggered proliferation and migration in SMCs, even in the presence of these mutations in the RAGE cytoplasmic domain^24^.

Experimental evidence suggests that the various ligands of RAGE bind to the extracellular domains of the receptor by distinct biophysical mechanisms. Park and colleagues demonstrated that recognition of the RAGE ligand S100B by RAGE occurs via an entropically-mediated process involving Ca^2+^-dependent hydrophobic interaction with the RAGE extracellular domains V-C1^7^. Koch and colleagues also identified the importance of RAGE V-C1 in binding to S100B^6^. However, Xie and colleagues demonstrated that a distinct S100, S100A12, binds to the C1-C2 domains of RAGE^25^ and Leclerc and colleagues showed that another S100 ligand of RAGE, S100A6, also binds to the C1-C2 extracellular RAGE domains^14^. In contrast, RAGE binding to AGEs is mediated by the recognition of negative charges displayed by the AGE-modified proteins. Xue and colleagues demonstrated that specific AGEs, carboxyethyllysine (CEL) and hydroimidazolone, fit into positively charged pockets within the V domain^8^,^26^. In the case of amyloid-ß-peptide, evidence suggests that the V domain is the principal recognition site for this ligand^27^,^28^. Taken together, these examples underscore the complexity of RAGE ligand binding to the extracellular domains of the receptor. Hence, we reasoned that it was essential to identify a distinct means of antagonizing the ligand-RAGE interaction. Because of the requirement to establish the veracity of the RAGE cytoplasmic domain binding to DIAPH1 as a key mechanism of RAGE signal transduction, taken together with the fact that extracellular domain inhibition of RAGE has not yet been shown to be fully safe and efficacious in the diverse patho-biological settings characterized by RAGE actions in human disease, we focused on targeting the ctRAGE-DIAPH1 interaction.

Given the complex, multi-ligand nature of ligands binding to the extracellular domains of RAGE, we sought instead to discover small molecule inhibitors of the interaction of the ctRAGE with DIAPH1. Although the development of inhibitors of protein-protein interaction (PPI) has been considered challenging, published evidence strongly supports robust interest in this class of drug targets, particularly since PPI are highly relevant in most biological processes^29^,^30^. Hence, we developed a high throughput screening assay with which we probed a small molecule library of 58,000 compounds. Of these molecules, 13 displayed dose-dependent inhibition of ctRAGE binding to DIAPH1 and, by NMR spectroscopy, demonstrated direct binding to the ctRAGE. These identified molecules inhibit RAGE ligand-triggered signal transduction in *in vitro* and *in vivo* experimentation. Overall, these data establish that the cytoplasmic domain of RAGE is a *bona fide* target for therapeutic intervention in RAGE-mediated pathologies and illustrate a novel mechanism for specific inhibition of RAGE signaling.

## Results

### High throughput screening and identification of small molecule inhibitors of ctRAGE interaction with DIAPH1

**Figure 1:**
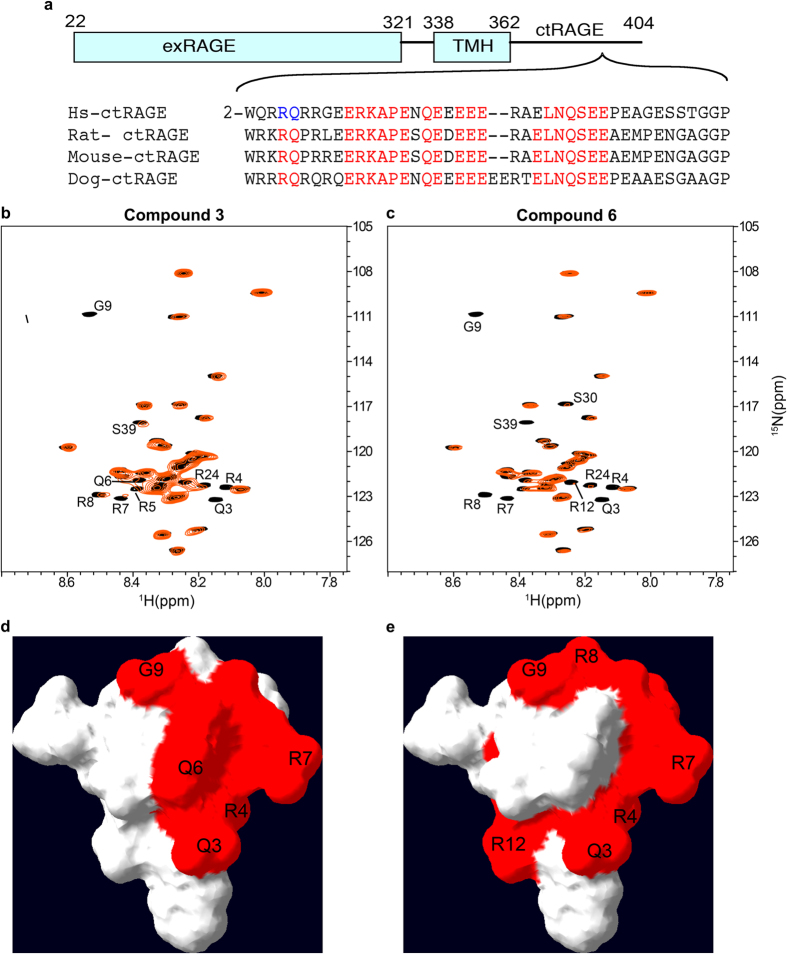
Selected library compounds bind to the structured part of ctRAGE. (**a**) Schematic representation of RAGE and the sequence alignment of ctRAGE. Homologous residues are in red. “TMH” indicates transmembrane helix and “exRAGE” indicates extracellular part of RAGE. Residues R5 and Q6, implicated in RAGE signaling, are in blue. (**b**,**c**) Overlay of the ^1^H{^15^N} HSQC NMR spectra of free [*U-*^15^N] ctRAGE (black) and [*U-*^15^N] ctRAGE bound to compound 3 (**b**) and 6 (**c**) (red). The NMR peaks of the ctRAGE residues that underwent extensive broadening due to interaction with the library compounds are labeled. (**d**,**e**) Interaction surface between ctRAGE and compounds 3 (**d**) and 6 (**e**). Residues Q3, R4, R5, and Q6 are previously implicated in binding of ctRAGE to DIAPH1.

Figure 1a illustrates the schematic representation of the human ctRAGE; the homologies among human, rat, mouse and dog ctRAGE are shown. To identify inhibitors of the human ctRAGE interaction with DIAPH1, we established a binding assay to probe ChemBridge’s 58,000 DIVERSet library, CT488A. The binding assay protocol and results are illustrated in Supplementary Table S1. Anti-DIAPH1 IgG was coated onto the wells of 384-well assay plates followed by blocking of unbound sites on the wells with bovine serum albumin (BSA). Following this step, the antibody-coated plates were incubated with human HeLa cell lysate (source of DIAPH1) followed by incubation with test small molecules (10 μM) and GFP-labeled ctRAGE. 777 small molecules that displayed ≥50% inhibition of specific binding were subjected to repetition and four point dose response (10 μM, 1 μM, 0.1 μM and 0.01 μM). In total, 97 compounds demonstrated dose-dependent inhibition of the binding of ctRAGE-DIAPH1. Supplementary Figure S1a–m demonstrates the results of the four point dose response study for compounds 1, 2, 3, 4, 5, 6, 7, 8, 9, 10, 11, 12, and 13 and identifies the compound structures. Note that compound 3, not a member of the initial library of compounds, is a close analogue of compound 4 and was purchased for additional support for this series after the primary screen.

Following these experiments, NMR spectroscopy was performed to determine the precise sites of interaction of these 97 compounds with the ctRAGE. Changes in protein NMR chemical shifts are exquisitely sensitive to the protein-ligand binding and are routinely used to identify protein-ligand interaction surfaces^31^. Based on the NMR experiments, thirteen compounds exhibited specific binding to ctRAGE. For example, ^1^H{^15^N} HSQC NMR spectra of [*U-*^15^N] ctRAGE reveal that both compounds 3 and 6 bind to the ctRAGE surface consisting of Q3, R4, R7, G9 and R12, which is similar to the RAGE cytoplasmic domain – DIAPH1 interaction site^24^, thus suggesting that both compounds directly interfere with this binding interaction (Compound 3, Fig. 1b,d and Compound 6, Fig. 1c,e). Of note, the ctRAGE interaction sites with compounds 3 and 6 do not completely overlap, as compound 6 also engages R8, S30 and S39. Further, compound 3 also engages R5 and Q6 of ct RAGE.

The NMR spectra of [*U-*^15^N] ctRAGE bound to the remaining eleven selected compounds are shown in the Supplementary Figures as follows: compounds 1, 2, 4, 5 (Supplementary Figure S2a–d); compounds 7, 8, 9 and 10 (Supplementary Figure S2e–h); and compounds 11, 12, and 13 (Supplementary Figure S2i–k). The interaction sites of ctRAGE with the remaining compounds do not completely overlap and also contain the residues involved in the ctRAGE-DIAPH1 interaction^24^. Taken together, these data indicate that from the original library, thirteen compounds were identified, which displayed nM dissociation constants for their specific binding to the RAGE cytoplasmic domain. Supplementary Table S2 summarizes these data.

**Figure 2:**
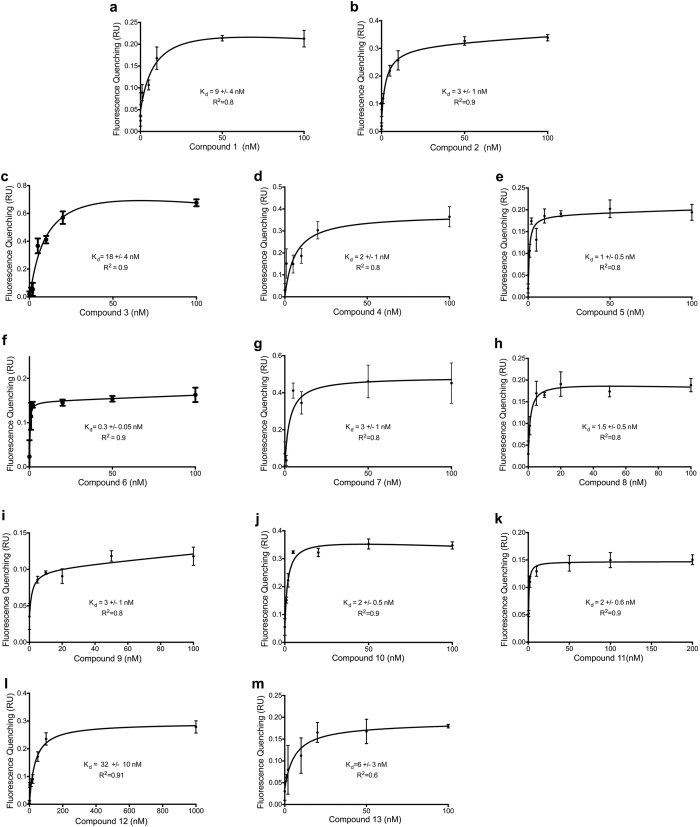
Small molecule competitive inhibition of binding of ctRAGE to DIAPH1. (**a–m**) Binding of compounds 1–13 to ctRAGE was monitored by native tryptophan fluorescence of ctRAGE. Dissociation constants (K_d_) are illustrated in the figure.

Native tryptophan fluorescence of ctRAGE was used to measure the binding affinity of the thirteen compounds that displayed dose-dependent inhibition of ctRAGE-DIAPH1 interaction and exhibited specific binding to ctRAGE. In these fluorescence titration experiments, dissociation constants, K_d_, were estimated from the changes in peak fluorescence intensities as a function of the free compound concentration and data were fit to the equation, (F − F_0_)/F_max_ = [compound]/(K_d_ + [compound]), where F is the fluorescence intensity at a given compound concentration, F_0_ is the fluorescence intensity of the blank, and F_max_ is the maximum fluorescence intensity. The thirteen compounds, illustrated in Fig. 2a–m, exhibited low nanomolar affinity for ctRAGE.

Based on these findings, we next sought to determine if the thirteen compounds affected the consequences of RAGE ligands in *in vitro* cellular assays and *in vivo.*

### Effects of compounds 1–13 on RAGE ligand-stimulated cellular signaling: Akt and ERK1/2

**Figure 3:**
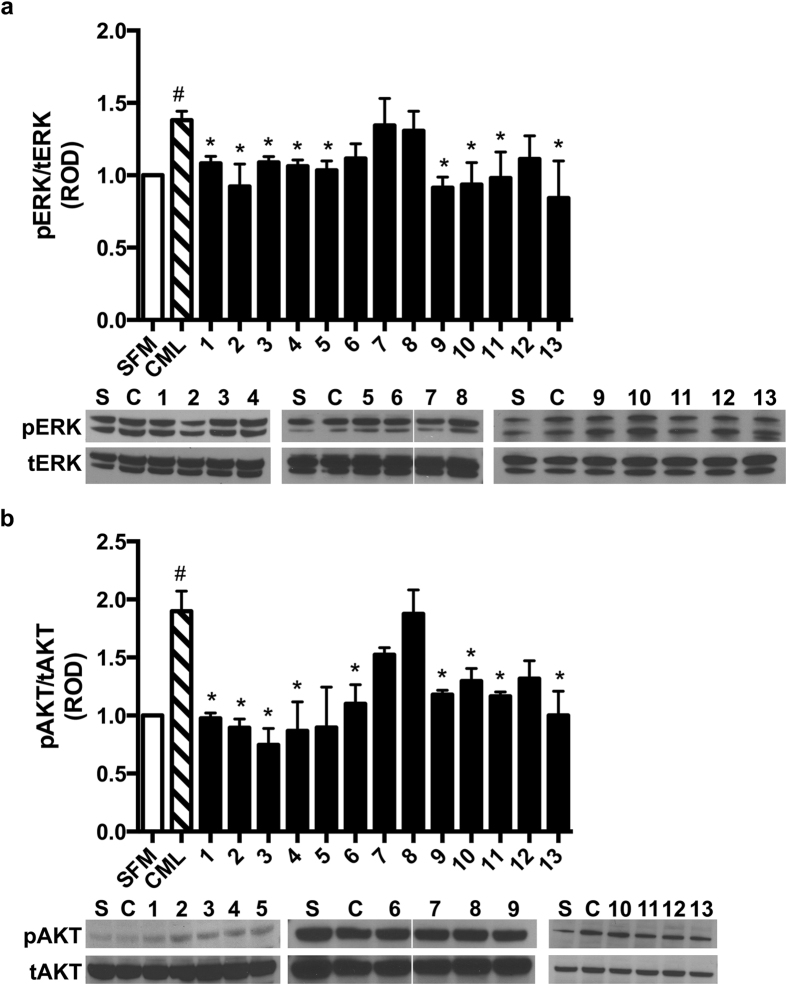
Effects of compounds 1–13 on RAGE ligand-stimulated phosphorylation of ERK and AKT in primary murine aortic SMCs. Primary murine SMCs were incubated with compounds 1–13 (1 μM) for 1.5 h and then treated with RAGE ligand CML-AGE (10 μg/ml) for 20 minutes. Cells were harvested and total lysates were subjected to Western blotting with antibodies against phospho-ERK and phospho-AKT. Blots were then re-probed with antibodies to total-ERK and total-AKT and relative optical density (ROD) is reported. (**a**) Quantified levels of phosphorylated/total ERK normalized to total ERK are shown. (**b**) Quantified levels of phosphorylated/total AKT normalized to total AKT are shown. Assays results shown are representative of three independent experiments. Error bars represent SEM. *indicates p < 0.05 comparing CML-AGE vs. CML-AGE and compound treatment and ^#^indicates p < 0.001 comparing CML-AGE vs. serum free medium (SFM) alone vehicle. Above each Western blot, S = serum-free medium and C = CML-AGE; numbers above the blots indicate the compound number.

We tested the effects of compounds 1–13 on cellular signaling in primary murine aortic SMCs. In SMCs, we previously showed that mutation of the two key amino acid residues of ctRAGE, R5/Q6, blocked cellular signaling induced by RAGE ligands. In primary murine aortic SMCs, we tested the effects of CML-AGE RAGE ligand on Akt and ERK1/2 phosphorylation, as these pathways are seminally linked to fundamental properties of these cells and their responses to stress, such as growth, proliferation, hypertrophy, migration, and survival^32^. As illustrated in Fig. 3a,b, compared to serum free medium (denoted as SFM or S in the figure), treatment of primary murine aortic SMCs with RAGE ligand CML-AGE (10 μg/ml) (denoted as CML or C in the figure) resulted in an 1.4- and 1.9-fold increase in phospho/total-ERK1/2 and phospho/total Akt; p < 0.001, respectively. Pretreatment of the cells for 1.5 h with compounds 1, 2, 3, 4, 5, 9, 10, 11, and 13, 1 μM, resulted in significant suppression of CML-AGE-mediated phospho/total ERK1/2. Only compounds 6, 7, 8, and 12 did not significantly affect ERK1/2 signaling (Fig. 3a). Pretreatment of the cells for 1.5 h with compounds 1, 2, 3, 4, 6, 9, 10, 11, and 13, 1 μM, resulted in significant suppression of CML-AGE-mediated stimulation of phospho/total-Akt (Fig. 3b). Only compounds 5, 7, 8, and 12 did not significantly suppress Akt signaling mediated by RAGE ligand (Fig. 3b).

### Effects of compounds 1–13 on RAGE ligand-stimulated smooth muscle cell migration

**Figure 4:**
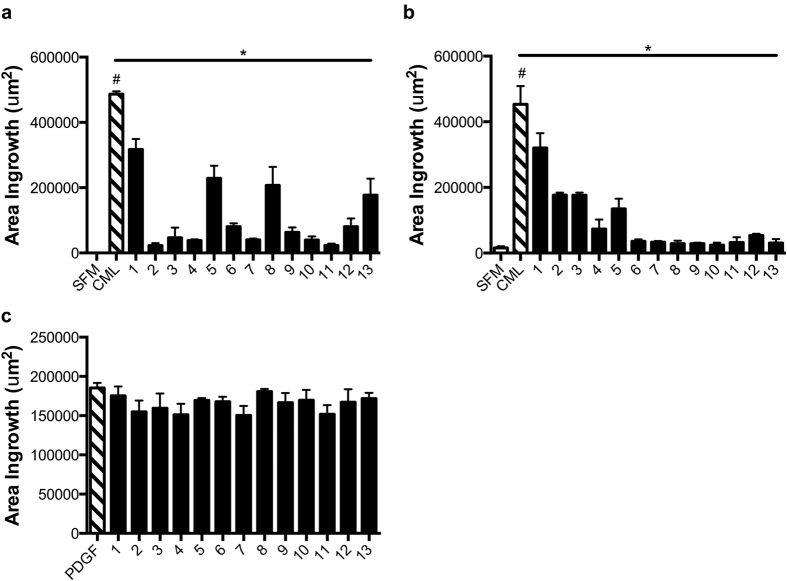
Effects of compounds 1–13 on RAGE ligand- or PDGF-stimulated migration of murine and human SMCs. Migration of cells after incubation with compounds 1–13 was assessed in murine SMC (**a,c**) and human SMC (**b**) migration. SMCs were grown to confluence and serum-starved overnight. The following morning, serum free media was removed and compounds 1–13 were added (1 μM). Immediately following the addition of compounds, the monolayer was wounded using a p200 pipette tip and allowed to incubate for 1.5 h. Following this incubation, compounds were removed and fresh media containing RAGE ligand, CML-AGE (10 μg/ml) (**a,b**) or general effector, PDGF-BB (10 ng/ml) (**c**), was added for 7 h. All images were measured at T0 and T7 h and an area ingrowth of effective migrating cells was calculated. Each compound was tested in three independent wells and each well was photographed in 3 separate locations for a total of 9 images used in assessing area ingrowth. Error bars represent SEM. *indicates p < 0.05 comparing CML-AGE vs. CML-AGE and compound treatment and ^#^indicates p < 0.001 comparing CML-AGE vs. serum free medium (SFM) vehicle.

Next, we tested the effects of the thirteen lead compounds on suppression of RAGE ligand-mediated cellular migration. To accomplish this, aortic SMCs were grown to confluence in cell culture-treated plates and then subjected to “wounding” using a single vertical scratch in the center of each well with p200 pipette tip. Immediately following the wounding, monolayers were pretreated with test compounds 1–13 for 1.5 h. Compounds were then removed from the monolayer and replaced with fresh medium containing the RAGE ligand CML-AGE (10 μg/ml). First, we studied the effects of the compounds in murine aortic SMCs. Compared to treatment of these cells with serum-free medium, treatment with RAGE ligand CML-AGE resulted in a significant increase in the area ingrowth (μm^2^); p < 0.001. Pretreatment with all of the compounds, 1–13, resulted in a statistically significant suppression of CML-AGE-mediated increased SMC ingrowth (Fig. 4a and Supplementary Figure S3a).

Next, we tested the effects of compounds 1–13 in human aortic SMC migration. Analogous studies to those performed in murine SMCs were performed. As shown in Fig. 4b, a significant increase in the area ingrowth upon treatment with RAGE ligand CML-AGE was observed vs. serum-free medium; p < 0.001. Pretreatment of human SMCs with all of the compounds, 1–13, resulted in a significant decrease in CML-AGE-mediated increased area ingrowth versus vehicle; p < 0.05 (Fig. 4b).

### Effects of compounds 1–13 on smooth muscle cell migration stimulated by non-RAGE ligand, PGDF-BB

Next, it was essential to test the effects of the compounds on the biological actions of a non-RAGE ligand. As discussed above, unlike treatment with the RAGE ligand S100B, in the presence of mutated R5/Q6 in the RAGE cytoplasmic domain, PDGF-BB stimulated significant migration and proliferation of murine SMCs^24^. To address this concept with the newly-identified small molecule inhibitors of the RAGE cytoplasmic domain interaction with DIAPH1, we tested the effects of compounds 1–13 on PDGF-BB-stimulated SMC migration. As illustrated in Fig. 4c and Supplementary Figure S3b, treatment with compounds 1–13 (1 μM), exerted no suppressive effect on PDGF-BB-stimulated murine SMC migration. Taken together, these data indicate that in SMCs, the small molecules 1–13 exhibit selectivity in inhibition of cellular migration induced by RAGE ligand CML-AGE, but not in response to non-RAGE ligand stimulus, PDGF-BB.

### Effects of compounds 1–13 on RAGE ligand-mediated stimulation of inflammation in endothelial cells and THP1 macrophage-like cells

In addition to potent effects on vascular cell migration, RAGE ligands are key mediators of inflammation in such settings as atherosclerosis, autoimmunity, diabetic complications, and in the central nervous system^17^,^33^,^34^,^35^,^36^,^37^,^38^,^39^,^40^. We thus tested the effects of compounds 1–13 on RAGE ligand-mediated inflammatory stimulation in endothelial cells and macrophage-like cells.

**Figure 5:**
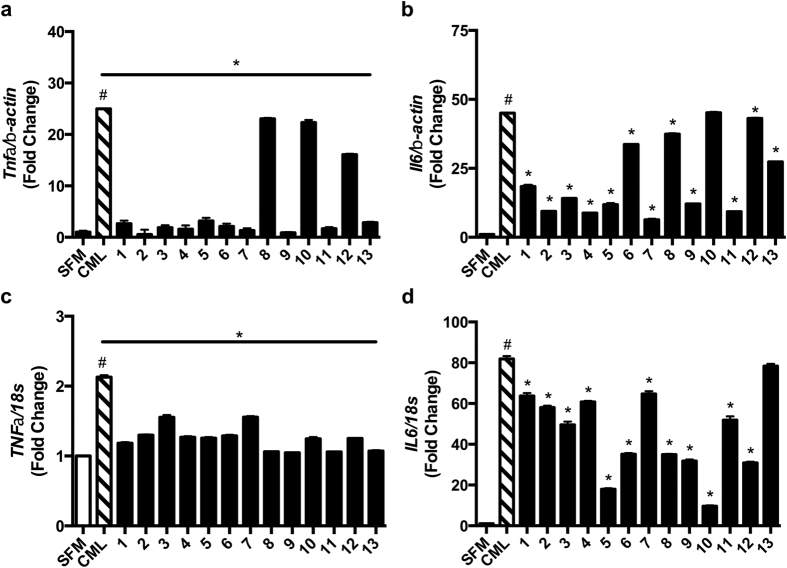
Effects of compounds 1–13 on RAGE ligand-stimulated inflammatory gene expression in primary murine aortic endothelial cells and human THP-1 cells. Primary murine aortic endothelial cells (**a,b**) or human THP1 cells (**c,d**) were subjected to 1.5 h incubation with compounds 1–13 (1 μM) followed by stimulation with CML-AGE (10 μg/ml) for 6 h. Cells were then harvested and gene expression of the inflammatory markers *Tnfα* and *Il6* normalized to β-actin (**a**,**b**) or 18s rRNA (**c,d**) was performed. Assays were performed in triplicate and results are representative of three independent experiments. Error bars represent SEM. *indicates p < 0.05 comparing CML-AGE vs. CML-AGE and compound treatment ^#^indicates p < 0.001 comparing CML-AGE vs. SFM vehicle (SFM).

First, we tested the effects of RAGE ligand CML-AGE in primary murine aortic endothelial cells (MAECs). Treatment with CML-AGE (10 μg/ml) resulted in a 25-fold and 45-fold increase in mRNA transcripts for the cytokines *Tnfa* and *Il6* compared to vehicle, SFM (Fig. 5a,b, respectively). To assess the effects of the compounds, we pre-treated MAECs for 1.5 h with compounds 1–13 (1 μM). Pretreatment with all thirteen compounds, 1–13, resulted in significant suppression of CML-AGE-mediated upregulation of *Tnfa* (Fig. 5a). Furthermore, pretreatment with compounds 1, 2, 3, 4, 5, 6, 7, 8, 9, 11, 12 and 13 resulted in significant suppression of CML-AGE-mediated upregulation of *Il6* (Fig. 5b).

Next, we tested the effects of CML-AGE in human macrophage-like THP1 cells on upregulation of inflammatory mediators. Compared to vehicle, SFM, CML-AGE stimulated a 2.2-fold and 82-fold increase in mRNA transcripts for the cytokines *Tnfa* and *Il6* (Fig. 5c,d, respectively). Analogous to experiments in MAECs, THP1 cells were pre-treated for 1.5 h with compounds 1–13 (1 μM) prior to the addition of RAGE ligands. Pretreatment with all thirteen compounds resulted in a significant suppression of CML-AGE-mediated upregulation of *TNFa* (Fig. 5c). Treatment with all but compound 13 resulted in significant suppression of CML-AGE-stimulated upregulation of *IL6* (Fig. 5d).

Taken together, these data indicate that small molecule inhibitors of the ctRAGE interaction with DIAPH1 suppress RAGE ligand-mediated inflammation in endothelial cells and THP1 macrophage-like cells.

### Effects of compounds on RAGE ligand-mediated stimulation of inflammation *in vivo*

**Figure 6:**
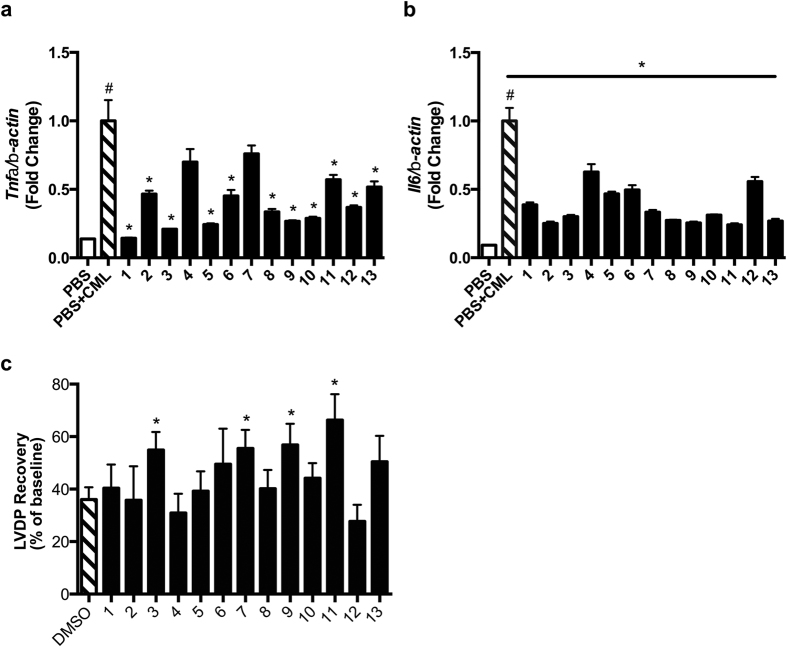
Effect of compounds 1–13 *in vivo.* (**a,b**) C57BL/6J mice were subjected to intraperitoneal administration of four doses of compounds 1–13 (5 mg/kg every 12 h) or equal volumes of vehicle (PBS). Thirty minutes after the final injection, mice were injected with CML-AGE (150 μg) by intraperitoneal administration. Four h later, mice were sacrificed and kidney was retrieved. Real time quantitative PCR was performed for detection of *Tnfa* (**a**) and *Il6* (**b**). (**c**) Assessment of compounds on cardiac functional recovery in diabetes. C57BL/6 mice were rendered type 1 diabetic with streptozotocin. After a mean range of 37–45 days of untreated hyperglycemia, mice were sacrificed and hearts rapidly retrieved for assessment of the efficacy of compounds 1–13 (1 μM) or vehicle, DMSO, on left ventricular developed pressure (LVDP) after ischemia. All hearts were subjected to 30 minutes of global ischemia followed by 60 min of reperfusion. Test compounds were introduced 10 minutes prior to the start of ischemia and continued throughout the reperfusion protocol. In (**a–c**), N = 5–6 mice per group. Error bars represent SEM. In (**a**–**c**), *indicates p < 0.05 vs. respective control, and in (**a,b**), ^#^indicates p < 0.001 comparing CML-AGE vs. PBS treatment.

Next, it was essential to test the ability of the thirteen compounds to block RAGE ligand-mediated inflammation *in vivo.* In our previous work, we used antibodies to RAGE to definitively illustrate that infusion of wild-type non-diabetic mice with AGEs upregulated *Il6* in vascular tissues, via RAGE^41^. To test this concept in the present study, we treated wild-type mice with CML-AGE (150 μg) by intraperitoneal injection after pretreatment with four doses of compounds 1–13 (5 mg/kg per dose, 12 h apart). Thirty minutes after the fourth dose of compound, mice were injected with CML-AGE and sacrificed 4 h later. Kidney tissue was retrieved and subjected to real-time quantitative PCR for detection of transcripts encoding inflammatory mediators. CML-AGE resulted in significantly increased mRNA transcripts for *Tnfa* and *ll6;* p < 0.001 (Fig. 6a,b). Pretreatment with compounds 1, 2, 3, 5, 6, 8, 9, 10, 11, 12, and 13 resulted in significant suppression of CML-AGE-mediated upregulation of *Tnfa* mRNA; p < 0.05 (Fig. 6a); and pretreatment with all compounds 1–13 resulted in significant suppression of *Il6* mRNA versus vehicle in the kidney after injection of RAGE ligand, CML-AGE; p < 0.05 (Fig. 6b).

### Effect of compounds on ischemia/reperfusion injury in the diabetic heart

Finally, we tested the effects of these compounds in settings in which RAGE ligands naturally accumulate, particularly the diabetic heart. Indeed, our previous work linked the RAGE pathway to injury in the diabetic heart; further, we identified that ischemia/reperfusion itself generates RAGE ligands^42^,^43^,^44^. To test this concept with our newly-identified small molecules, we rendered wild-type mice diabetic with streptozotocin. After a mean range of 37–45 days of sustained, untreated hyperglycemia, hearts were retrieved and subjected *ex vivo* to ischemia/reperfusion injury. It is well-established that in this setting, diabetic hearts sustain greater injury compared to non-diabetic hearts^45^. In our experiments, we infused vehicle (DMSO) versus compounds 1–13 (final concentration, 1 μM) beginning ten minutes prior to the start of ischemia and continued throughout the 1 h ischemia/reperfusion period. At the end of reperfusion, we measured left ventricular developed pressure (LVDP) in the hearts, a key measure of functional recovery after injury. As illustrated in Fig. 6c, compared to DMSO vehicle, in which we observed a 36% functional recovery, treatment with compounds 3, 7, 9, and 11 resulted in a statistically-significantly higher LVDP, 55, 57, 56, 57 and 66%, respectively; p < 0.05. In no case did any of the compounds significantly reduce LVDP recovery. Of note, no differences were observed in the levels of glucose or body weight among the groups (Supplementary Table S3).

## Discussion

Despite extensive evidence that RAGE-dependent modulation of cellular properties was dependent on stimulation of signal transduction, the proximate mechanisms underlying RAGE signaling were only recently uncovered. Hence, the discovery that ctRAGE bound DIAPH1 and that DIAPH1 was required for the effects of RAGE ligand stimulation in vascular and immune cells set the stage for the discovery of an entirely novel class of RAGE pathway antagonists. In the current study, to summarize; we performed a high throughput binding assay to screen a 58,000 compound small molecule library for the identification of inhibitors of the interaction of ctRAGE with DIAPH1. Through a series of dose response, NMR spectroscopy and fluorescence titration experiments, we identified thirteen “hit” compounds that exhibited nM affinity binding to ctRAGE and that interfered with the ctRAGE-DIAPH1 complex. In *in vitro, ex vivo* and *in vivo* studies, these compounds displayed inhibitory effects on RAGE signal transduction, cellular migration, inflammatory gene expression and ischemia-induced perturbation of heart function in the isolated perfused diabetic heart. Notably, the non-overlapping binding sites of the compounds on ctRAGE may underscore the differences in their ability to interfere with RAGE function (Fig. 1d,e). Although previous studies have suggested that ctRAGE may bind to distinct intracellular effector molecules, such as extracellular related kinase (ERK) or toll-interleukin 1 receptor (TIR) adaptor protein (TIRAP)^46^,^47^, no inhibitors of the interaction of these intracellular targets with ctRAGE have yet to demonstrate *in vivo* evidence of suppression of RAGE ligand-mediated cellular stimulation, as illustrated herein. A recent report described the generation of ctRAGE inhibitor peptide; however, the peptide efficacy was only studied in the context of suppressing signal transduction in *in vitro* analyses^48^.

Previous studies have concluded that ctRAGE bears intrinsic flexibility^24^,^49^, thereby underscoring the structural diversity observed among the thirteen small molecule compounds identified from the 58,000 compound library. Our present results further validate the notion that flexible parts of a protein can serve as a valid therapeutic target^50^. It is notable that among these thirteen compounds, structural similarities amongst some of them have led to their assignment into distinct “lead series” versus single structurally unique “singleton” ctRAGE-DIAPH1 inhibitor analogs.

Likely vulnerabilities in the compounds identified from the original library will require mitigation in future studies, such as enhancement of solubility, permeability, stability, plasma and metabolic stability and *in vivo* half-life, as well as reduction of potential toxicities due to unknown off-target activities. Although no overt toxicities in cellular models or *in vivo* were noted in cells or animals treated with the small molecule compounds 1–13, it is possible that long-term administration would evoke possible side-effects. In this context, it is also possible that liabilities in solubility and permeability, in particular, limited effectiveness for some compounds *in vitro* and *in vivo.* Future directions actively underway include rigorous advancement of the structure activity relationships of the chemically-distinct series in order to optimize efficacy and physical characteristics of the compounds requisite for druggability.

Targeting ctRAGE-DIAPH1 interactions involves the development of PPIs. Unlike distinct peptide-based PPI inhibitors, in which immunogenicity and proteolytic digestion of the agents may represent a substantial impediment^51^, particularly in inflamed diseased environments, the current approach suggested herein employs small molecule compounds as putative inhibitors of ctRAGE interaction with DIAPH1. Indeed, Sheng and colleagues recently reviewed the state of this field^52^ and highlighted a number of recent clinical trials, which unveiled the druggability of small molecule PPI inhibitors in targeting cancer or ocular disorders. In those successful examples, the small molecule PPI inhibitors bore comparable affinity for natural protein binders.

The rationale for blockade of RAGE signal transduction is supported by work in a variety of preclinical disease models such as diabetes and its complications, Alzheimer’s disease^53^,^54^, cardiovascular disease^42^,^44^,^55^,^56^, immune/inflammatory disorders^57^,^58^ and cancer^59^,^60^, as examples. RAGE ligands accumulate in these settings and therefore their contribution to the pathogenesis of cellular dysfunction and tissue damage in these diseases may ultimately be mitigated by modulation of RAGE signaling.

Finally, we note that therapeutic opportunities regarding the pathobiology of RAGE have captured significant attention in the scientific community. In addition to strategies targeting direct antagonism of RAGE through blockade of ligand binding to the RAGE extracellular V-type domain, such as azeliragon (in Phase 3 clinical trials)^61^ and FPS-ZM1^27^, other approaches such as soluble RAGE-type molecules may be of benefit. In the latter case, it is postulated that administration of soluble (s) RAGE sequesters RAGE ligands, thereby preventing these ligands from interacting with the cell surface receptor^38^,^62^. It must be noted, however, that just as the receptor is promiscuous, so, too, are the ligands. The possibility that sRAGE modulates adaptive functions of a subset of these RAGE ligands in their binding to distinct beneficial cell surface receptors, needs to be addressed in future studies. These considerations notwithstanding, evidence is building that suggests the accumulation of sRAGEs in human subject plasma/serum, if not therapeutic moieties, may, indeed, be useful biomarkers of RAGE activity and cellular perturbation^63^,^64^. The extent to which inhibition of RAGE signal transduction, such as the approach postulated herein, as well as combination strategies for these RAGE-directed approaches, remains to be determined and continues to be the subject of active, ongoing investigation.

In summary, the feasibility of targeting RAGE signal transduction is evidenced by the following considerations: *first,* our published data indicate that the interaction surface of RAGE with DIAPH1 is small, less than 200 Å^2^, and, thus targetable by a small molecule strategy; *second*, despite caveats in terms of solubility, permeability, stability and half-life, many of the thirteen compounds identified from the original library, which demonstrated nM affinity to ctRAGE, demonstrated efficacy in blocking RAGE ligand-, but not RAGE-independent stimuli-mediated function. We conclude that these identified compounds hold significant potential as druggable scaffolds for further development for the treatment of RAGE-related disorders.

## Methods

### High Throughput Screening Assay

Anti-DIAPH1 antibody (Santa Cruz Biotechnology, Dallas TX), 62.5 ng/well, was prepared as a 1:160 dilution in 0.1M Na_2_CO_3_ pH = 9.6; final volume, 0.05 ml, and coated onto the wells of 384-well plates for 16 h at 4 °C. Plates were washed with PBS, followed by blocking with bovine serum albumin (BSA) (3%) (0.1 ml/well) for 1.5 h at room temperature. This was followed by washing with PBS and the addition of human HeLa cell lysate (DIAPH1 source), 10 μg/well, for 3 h at room temperature. Wells were washed with PBS followed by treatment for 2 h with the test small molecules of Chembridge’s DIVERSet Ct488 library (final concentration, 10 μM) and GFP-ctRAGE (final concentration, 64.5 μM) for 2 h at room temperature. Test compounds were added from the original library plates using the JANUS Automated Workstation (Perkin Elmer, Waltham, MA). GFP-ctRAGE binding to DIAPH1 was determined in a EnVision Multilabel Reader (Perkin Elmer, Waltham, MA) and results normalized as described in Supplementary Table S1. All plate washes were performed using Model Elx405 (Bio Tek, Winooski, VT).

### Fluorescence titration experiments

Native tryptophan fluorescence experiments were conducted using a Horiba Jobin Yvon Fluorolog spectrofluorometer. 97 library compounds that passed the ELISA screening were used in the fluorescence titrations experiments. 1 mM of the library compound was dissolved in 10 mM phosphate buffer [pH 7.0] and 50% DMSO. ctRAGE was bacterially expressed and purified as described^24^. 10 nM ctRAGE solution was individually titrated from 0.1 nM–100 μM with the compounds in 100 μL of 10 mM phosphate buffer [pH 7.0] and 5% DMSO. The excitation and emission wavelengths were 280 nm and 352 nm, respectively. Dissociation constants, K_d_, were estimated from the changes in peak fluorescence intensities as a function of the free compound concentration by using Prism 5 software (GraphPad). Data were fit to the equation, (F − F_0_)/F_max_ = [compound]/(K_d_ + [compound]) where F is the fluorescence intensity at a given compound concentration, F_0_ is the fluorescence intensity of the blank, and F_max_ is the maximum fluorescence intensity.

### NMR Spectroscopy

NMR experiments were performed on Bruker Avance III spectrometers equipped with a cryoprobe, operating at ^1^H frequencies of 500 MHz and 700 MHz. 97 library compounds that passed the ELISA screening were used in the NMR experiments. NMR samples contained 50 μM of [*U-*^15^N] RAGE tail and 10 μM of the library compound in 10 mM phosphate buffer [pH 7.0] and 5% d_6_-DMSO. All spectra were collected at 298 K, which yielded high quality NMR spectra of [*U-*^15^N] ctRAGE^24^. We used a Watergate version of the ^1^H{^15^N}- edited heteronuclear single quantum coherence (HSQC) experiment^65^ recorded with 64 transients as 512 × 64 complex points in proton and nitrogen dimensions, respectively, apodized with a squared cosine-bell window function and zero-filled to 1024 × 128 points prior to Fourier transformation. The corresponding sweep widths were 12 and 35 ppm in the ^1^H and ^15^N dimensions, respectively. The spectra were processed by using the program TOPSPIN 2.1 (Bruker, Inc) and the program CARA^66^ was used for spectral analysis. Chemical shifts [*U-*^15^N] ctRAGE were assigned^24^. To reassign the [*U-*^15^N] RAGE tail peaks that changed position due to complex formation we assumed minimum chemical shift changes^67^, calculated as ΔΩ = ((ΔΩ_HN_)^2^ + (0.25 * ΔΩ_N_)^2^)^1/2^, where Ω_NH_ and Ω_N_ represent amide hydrogen and nitrogen chemical shifts, respectively. Peak intensity changes were calculated as: (I/I_ref_)_free_ − (I/I_ref_)complex, where *I* is an individual peak intensity and I_ref_ is the peak intensity of a glutamine at 7.45 ppm and 112.5 ppm in the proton and nitrogen dimensions, respectively, that does not shift during titration.

### Western Blotting

Total protein extracts were prepared from primary murine aortic smooth muscle cells using cell lysis buffer (Cell Signaling Technology, Beverly, MA). Equal amounts of protein (30 μg/sample) were subjected to SDS-PAGE (4–12%) followed by electrophoretic transfer to nitrocellulose membranes. Nonspecific binding was blocked by incubation of membranes with BSA (5%) for 1 h. The blot was incubated with one of the following antibodies as the primary antibody for the reaction: anti-phospho-ERK1/2 (P-ERK1/2) IgG, anti-total-ERK1/2 (T-ERK1/2) IgG, anti-phospho-AKT (P-AKT) IgG, and anti-total-AKT (T-AKT) IgG (Cell Signaling Technology). Each primary antibody was used at a dilution of 1:1,000 overnight at 4 °C according to the manufacturer’s instructions. Horseradish peroxidase-conjugated secondary antibodies (1:2,500; Amersham Biosciences) were used to identify sites of binding of each primary antibody.

### Cell culture and *in vitro* assays on cultured SMCs

Murine vascular SMCs were cultured from the aortas of 10-week old male mice using a modification of the procedure of Travo and Barret^68^. SMCs were cultured following an explant protocol in accordance with institutional guidelines. Wild-type murine aortic vascular SMCs were isolated and used between passages 8 to 12. Human aortic vascular smooth muscle cells were purchased from American Type Culture Collection (ATCC, Manassas, VA) and cultured according to the methods recommended by ATCC. Murine primary aortic endothelial cells were from 6- to 8-week-old wild-type C57BL/6J mice (Jackson Laboratories, Bar Harbor ME) and used under passage 10^69^. THP-1 cells were obtained from the American Type Tissue Collection (ATCC, Manassas VA) and used according to the manufacturer’s instructions.

### Real-Time Quantitative PCR

Total RNA was extracted from cells and tissues using the RNeasy lipid kit (Qiagen, Hilden, Germany). cDNA was synthesized with MultiScribe reverse transcriptase (Applied Biosystems, Foster City, CA). Real-time quantitative PCR was performed using the TaqMan method (50 °C for 2 min, 95 °C for 10 min, and 40 cycles of 95 °C for 15 s and 60 °C for 1 min) with premade primer sets (Applied Biosystems). The relative abundance of transcripts was normalized according to the expression of 18S rRNA (for human cells) or β-actin (for murine cells and tissues) using the ΔΔCt method. Supplementary Table S4 lists the specific primers used in the outlined studies.

### Smooth Muscle Cell Migration

Migration in response to the RAGE ligand CML-AGE (CML, 10 μg/ml) or a general effector, not a RAGE ligand, PDGF-BB, 10 ng/ml (R&D systems, Minneapolis, MN, USA) was assessed with a wounding assay. Cells were grown to confluence in 12-well plates and starved overnight. The following morning, serum free media was removed and compounds 1–13 were added. Immediately following the addition of compounds, the monolayer was wounded using a p200 pipette tip and the compounds were allowed to incubate for 1.5 h. Following this incubation, all compounds were removed and fresh media containing RAGE ligand or general effector, PDGF-BB, was added for 7 h. Cells were maintained at 37 °C and 5% CO_2_. Images were obtained at T0 and T7. Each image was measured and an area ingrowth of effective migrating cells was calculated.

### Animal studies

All animal procedures were approved by the Institutional Animal Care and Use Committees of Columbia University and New York University and performed in accordance with the National Institutes of Health Animal Care Guidelines. Specific experiments in mice are described below.

### Isolated perfused heart and measurement of cardiac function

Male C57BL/6 mice, age 8–12 weeks, were purchased from The Jackson Laboratory and rendered diabetic by 55 mg/kg by intraperitoneal route of streptozotocin (STZ) (Sigma Aldrich, St. Louis MO) per day in fresh citrate buffer (0.05 mol/l; pH 4.5) for 5 consecutive days. Control mice received citrate buffer alone. Mice displaying serum glucose ≥250 mg/dl were considered diabetic. Animals were sacrificed over a mean range of 37 and 45 days of diabetes. Experiments were performed using an isovolumic mouse heart preparation as we previously published^70^,^71^. After deep anesthesia was achieved, hearts were rapidly excised, placed in an iced cold Krebs-Henseleit buffer and then retrogradely perfused with modified Krebs-Henseleit buffer containing (in mM) NaCl 118, KCl 4.7, CaCl_2_ 2.5, MgCl_2_ 1.2, NaHCO_3_ 25, glucose 5, palmitate 0.4, bovine serum albumin 0.4, and 70 mU/L insulin, at 37 °C in a non-recirculating mode through the aorta. The perfusate was equilibrated with a mixture of 95% O_2_-5% CO_2_, which maintained perfusate PO_2_ > 600 mm Hg. Left ventricular developed pressure (LVDP) was measured using a latex balloon in the left ventricle and monitored continuously on an ADI recorder. All mouse hearts were subjected to 30 minutes of baseline monitoring followed by 30 minutes of zero-flow ischemia and 60 minutes of reperfusion (I/R). Test compounds were introduced 10 minutes prior to the start of ischemia and continued throughout the reperfusion protocol. Note that upon confirmation of diabetes, mice were randomly assigned to the treatment group. Supplementary Table S3 indicates the levels of blood glucose, body weight, and days of diabetes for all mice in the assigned compound treatment vs. vehicle groups.

### Infusion of CML-AGE *in vivo* and the effects of compounds 1–13

Male, wild-type C57BL/6J mice were purchased from the Jackson Laboratories. At age 8 weeks, mice were pretreated with 4 doses of compounds 1–13, 5 mg/kg, by intraperitoneal administration twice daily for a total of two days and four total doses. Control treatment consisted of DMSO diluted into PBS. Thirty minutes after the last dose, CML-AGE (150 μg) was injected by intraperitoneal route. 4 h later, mice were sacrificed and kidney tissue was rapidly retrieved for preparation of mRNA and performance of real time quantitative PCR for the following mRNA transcripts, *Tnfa* and *Il6.*

### Statistical Analysis

All data are expressed as the mean ± SEM. Statistical significance was analyzed by unpaired two-tailed t-test using GraphPad Prism version 6.0f for MacOS X, GraphPad Software, La Jolla CA. *p* values < 0.05 were considered statistically significant.

## Additional Information

**How to cite this article**: Manigrasso, M. B. *et al.* Small Molecule Inhibition of Ligand-Stimulated RAGE-DIAPH1 Signal Transduction. *Sci. Rep.* **6**, 22450; doi: 10.1038/srep22450 (2016).

## Supplementary Material

Supplementary Information
