# Supplementary material for: Small Molecule Inhibition of Ligand-Stimulated RAGE-DIAPH1 Signal Transduction
Source: Sci Rep. 2016 Mar 3;6:22450. doi: 10.1038/srep22450 (PMC4776135; doi:10.1038/srep22450)
Supplement: Supplementary Information [file srep22450-s1.pdf]

Supplementary Information for:

Small Molecule Inhibition of Ligand-Stimulated RAGE-DIAPH1 Signal Transduction

Michaele B. Manigrasso[1@], Jinhong Pan[2@], Vivek Rai[1,@], Jinghua Zhang[1], Sergey Reverdatto[2], Nosirudeen Quadri[1], Robert J. DeVita[3], Ravichandran Ramasamy[1], Alexander Shekhtman[2,^*] & Ann Marie Schmidt[1^*]

@ These authors contributed equally to this work.

^Co-Senior Authors

[1]Diabetes Research Program, Department of Medicine, New York University Langone Medical Center, 550 First Avenue, New York, New York 10016

[2]Department of Chemistry, University at Albany, State University of New York,

1400 Washington Avenue, Albany, New York 12222

[3]RJD Medicinal Chemistry and Drug Discovery Consulting LLC, 332 W. Dudley Avenue, Westfield, New Jersey 07090

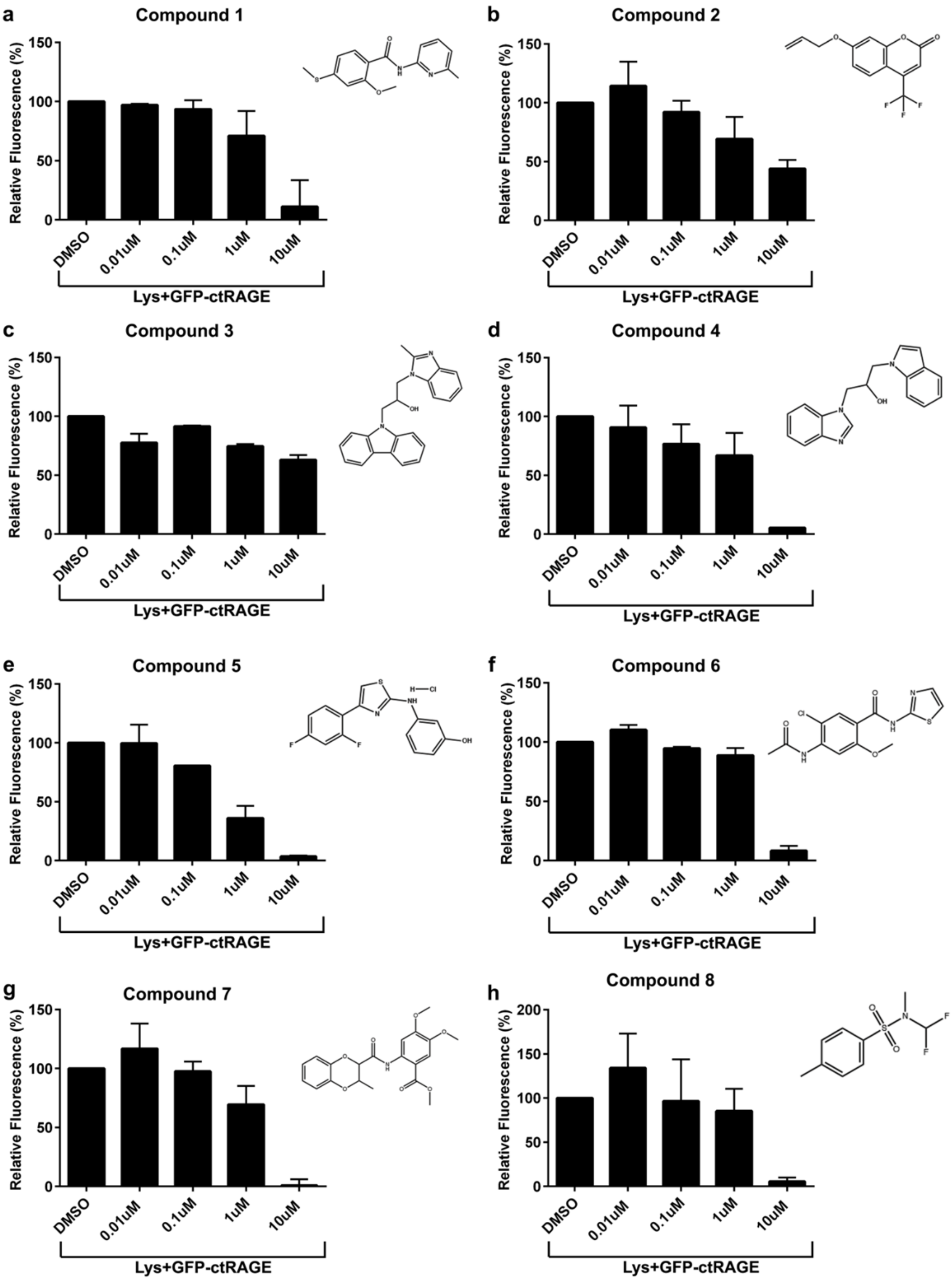

a
Compound 1
Relative Fluorescence (%)
0
50
100
150
DMSO
0.01uM
0.1uM
1uM
10uM
Lys+GFP-ctRAGE
b
Compound 2
Relative Fluorescence (%)
0
50
100
150
DMSO
0.01uM
0.1uM
1uM
10uM
Lys+GFP-ctRAGE
c
Compound 3
Relative Fluorescence (%)
0
50
100
150
DMSO
0.01uM
0.1uM
1uM
10uM
Lys+GFP-ctRAGE
d
Compound 4
Relative Fluorescence (%)
0
50
100
150
DMSO
0.01uM
0.1uM
1uM
10uM
Lys+GFP-ctRAGE
e
Compound 5
Relative Fluorescence (%)
0
50
100
150
DMSO
0.01uM
0.1uM
1uM
10uM
Lys+GFP-ctRAGE
f
Compound 6
Relative Fluorescence (%)
0
50
100
150
DMSO
0.01uM
0.1uM
1uM
10uM
Lys+GFP-ctRAGE
g
Compound 7
Relative Fluorescence (%)
0
50
100
150
DMSO
0.01uM
0.1uM
1uM
10uM
Lys+GFP-ctRAGE
h
Compound 8
Relative Fluorescence (%)
0
50
100
150
200
DMSO
0.01uM
0.1uM
1uM
10uM
Lys+GFP-ctRAGE

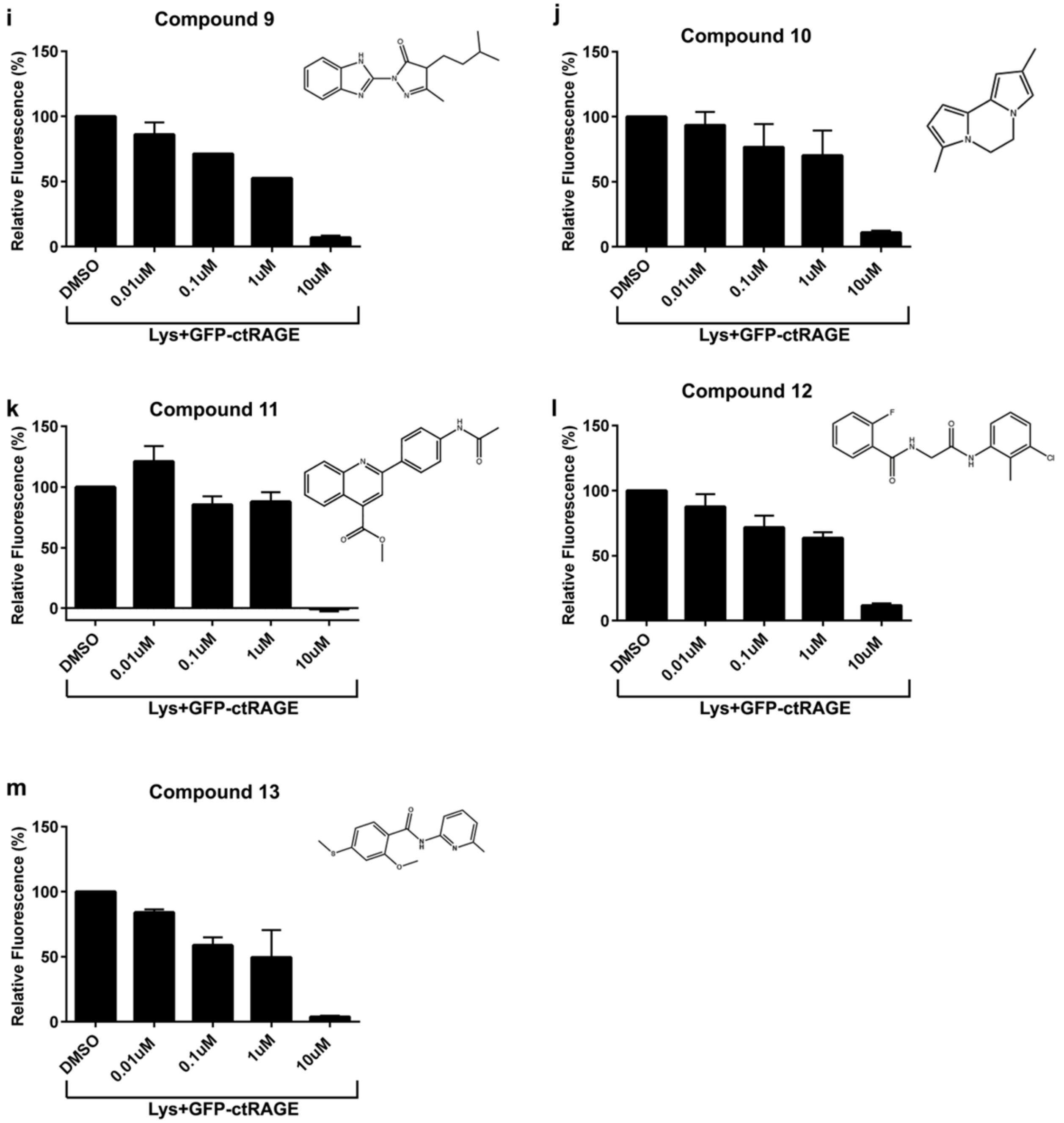

Supplementary Figure S1. Small molecule competitive inhibition of binding of ctRAGE to DIAPH1. (a-m). Testing of compounds 1-13 in the high throughput screening assays reveals a dose-dependent binding of fluorescent GFP-labeled ctRAGE to DIAPH1. Compounds were tested in duplicate at 10 µM, 1 µM, 0.1 µM, and 0.01 µM. Structures of compounds 1-13 are illustrated in the figure.

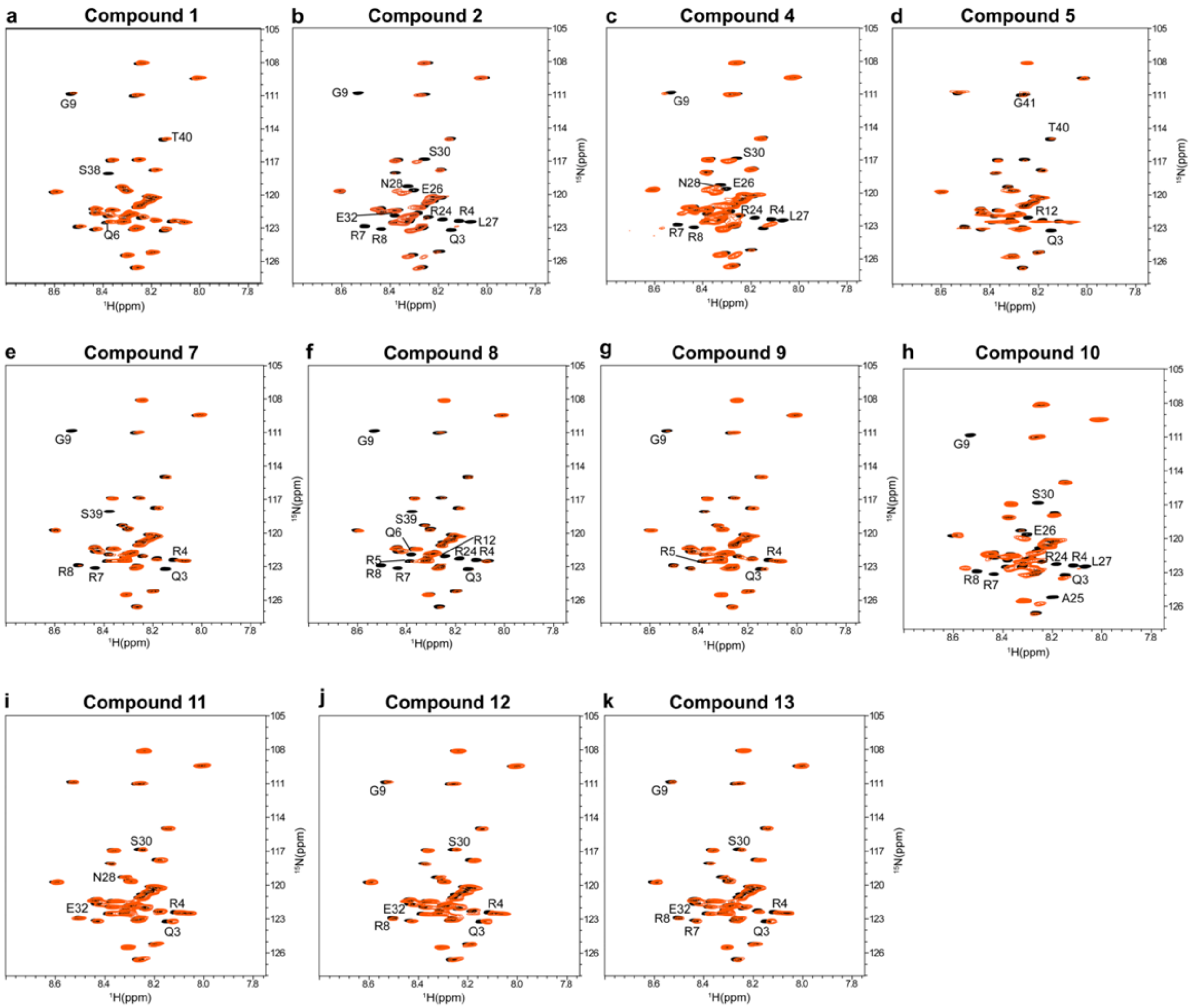

Supplementary Figure S2. Compounds 1, 2, 4, 5, 7, 8, 9, 10, 11, 12, and 13 bind to the structured part of ctRAGE. $^{1}H\{^{15}N\}$ HSQC NMR spectra of [*U*-^{15}N] RAGE tail bound to compound 1(a), 2(b), 4(c), 5(d), 7(e), 8(f), 9(g), 10(h), 11(i), 12(j), and 13(k). The NMR peaks of ctRAGE residues that underwent either changes in the chemical shifts or extensive broadening due to interaction with the library compounds are labeled.

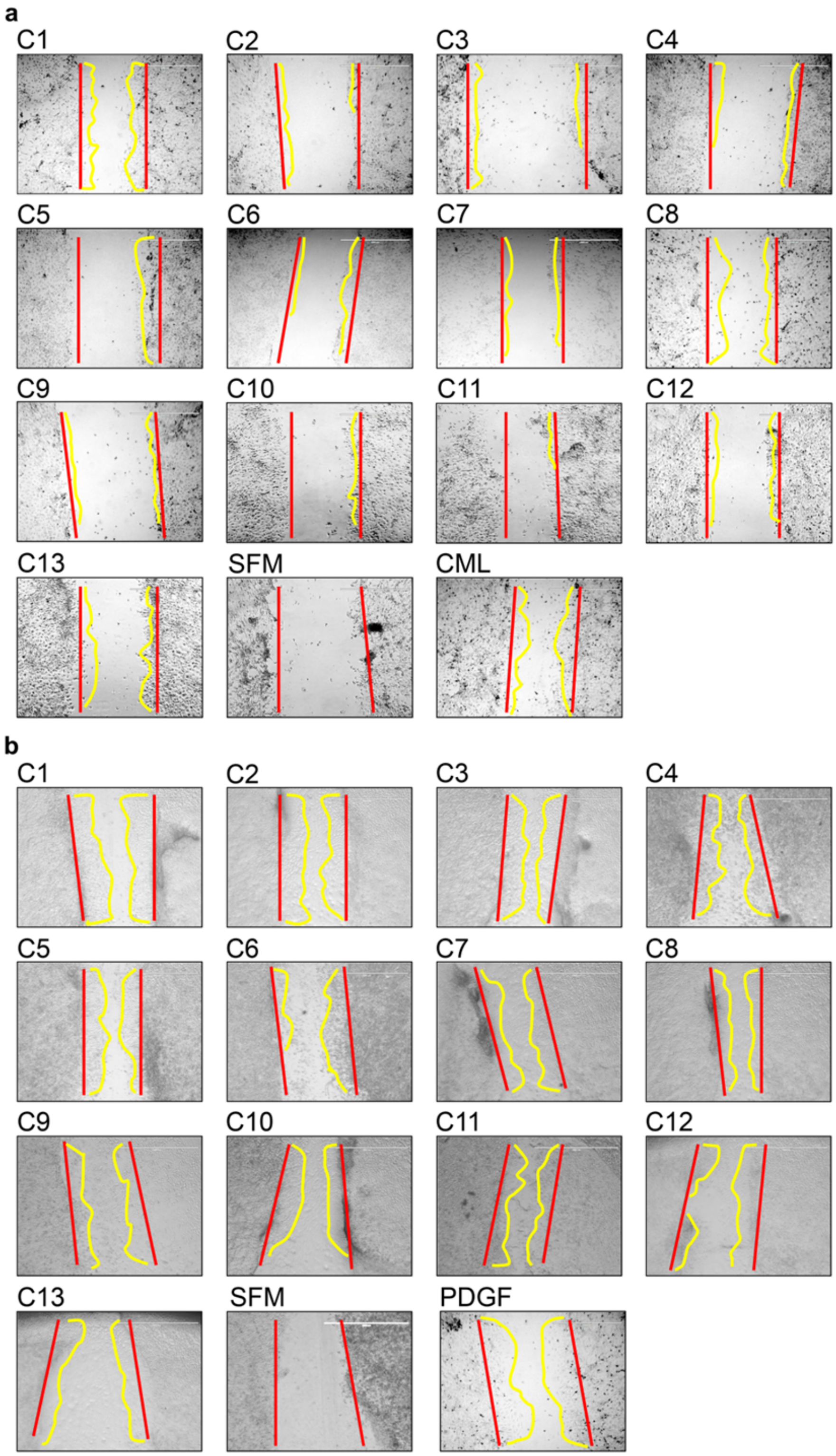

Supplementary Figure S3. Effects of compounds 1-13 on RAGE ligand- or PDGF-stimulated migration of murine SMCs. Migration of SMCs after incubation with compounds

1-13 was assessed. After the addition of the compounds 1-13 (1 μM) to the individual wells, a wound was immediately created down the center of the well using a p200 tip and a baseline photograph was taken. After 1.5 h incubation, the compounds were removed and murine aortic SMCs were stimulated with CML-AGE (10 μg/ml) (a) or PDGF-BB (10 ng/ml) (b) for 7 h, and final images were taken. Each compound was tested in three independent wells and each well was photographed in 3 separate locations for a total of 9 images used. Representative images are shown.

Supplementary Table S1. Small molecule screening protocol and results

Category	Parameter	Description
Assay	Type of assay	*In vitro* Binding Assay
	Target	GFP-ctRAGE – DIAPH1 binding
	Primary measurement	GFP Fluorescence
	Key reagents	GFP-ctRAGE and DIAPH1 (HeLa cell lysate)
	Assay protocol	High Throughput Binding Assay (See Methods)
	Additional comments	N/A
Library	Library size	58,000 compounds
	Library composition	Small molecule commercially-available library
	Source	DIVERSet library (CHEMBRIDGE), CT488A
	Additional comments	N/A
Screen	Format	384 well format
	Concentration(s) tested	10 µM, final concentration dissolved in 0.1% DMSO
	Plate controls	Hela Lysate (DIAPH1) and GFP-ctRAGE
	Reagent/ compound dispensing system	Microplate Reader (PerkinElmer, Inc)
	Detection instrument and software	Envision Software
	Assay validation/QC	Z' score, 0.62; S/N, 8.3
	Correction factors	None
	Normalization	Plate background with no GFP
	Additional comments	N/A
Post-HTS analysis	Hit criteria	>50 % inhibition of binding of ctRAGE to DIAPH1
	Hit rate	1.5 %
	Additional assay(s)	Secondary Assay – Positive hits above (N=777) subjected to 4 point dose response [10, 1, 0.1 and 0.01 µM] → (N=97) Compounds Subjected to Nuclear Magnetic Resonance (NMR) for Binding to ctRAGE and not DIAPH1 → (N=13) Subjected to Fluorescence Titration for IC50
	Confirmation of hit purity and structure	Liquid chromatography-Mass Spectrometry and NMR
	Additional comments	N/A

Supplementary Table S2. Compounds 1-13 Bind to the Cytoplasmic Domain of RAGE: Dissociation Constants

Compound	Dissociation Constant (nM)
1	9±4
2	3±1
3	18±4
4	2±1
5	1±0.5
6	0.3±0.05
7	3±1
8	1.5±0.5
9	3±1
10	2±0.5
11	2±0.6
12	32±10
13	6±3

Supplementary Table S3. Characteristics of Mice Studied in the Isolated Perfused Heart

	**DMSO**	**C1**	**C2**	**C3**	**C4**	**C5**	**C6**
**n**	6	6	6	6	6	5	6
**Body Weight (g)**	25.2±2.7	24.4±3.1	22.4±4.4	25.1±2.8	24.8±3.7	25.6±3.6	21.9±1.9
**Glucose (mg/dL)**	333±76	367±83	357±58	362±65	379±74	285±40	347±63
**Days diabetic**	40±10	44±5	44±5	39±7	42±7	41±8	38±11
	**C7**	**C8**	**C9**	**C10**	**C11**	**C12**	**C13**
**n**	6	6	6	6	6	6	6
**Body Weight (g)**	21.6±1.4	23.67±1.9	25.1±5.0	24.2±2.9	25.1±2.4	24.8±2.3	24.0±3.0
**Glucose (mg/dL)**	333±77	329±73	328±43	380±68	348±46	353±86	327±43
**Days diabetic**	45±7	38±7	40±6	37±8	40±6	42±7	38±9

Supplementary Table S4. Materials for performance of the real time PCR experiments

Gene Name (Taqman)	Gene Symbol	Assay ID/catalog number
TNF	*Tnf*	Mm00443258_m1
IL6	*Il6*	Mm00446190_m1
ACTB	*Actb*	4352341E
TNF	*TNF*	Hs01113624_g1
IL6	*IL6*	Hs00985639_m1
Eukaryotic 18S	*18S*	4310893E
